# Down-regulation of G9a triggers DNA damage response and inhibits colorectal cancer cells proliferation

**DOI:** 10.18632/oncotarget.2784

**Published:** 2015-01-20

**Authors:** Jie Zhang, Pengxing He, Yong Xi, Meiyu Geng, Yi Chen, Jian Ding

**Affiliations:** ^1^ Division of Anti-Tumor Pharmacology, State Key Laboratory of Drug Research, Shanghai Institute of Materia Medica, Chinese Academy of Sciences, Shanghai 201203, China; ^2^ School of Pharmaceutical Sciences, Zhengzhou University, Zhengzhou 450001, China

**Keywords:** colorectal cancer, G9a, epigenetics, DNA damage response (DDR), SN38/CPT, synergistic effect

## Abstract

G9a, a histone methyltransferase, is aberrantly expressed in some human tumor types. By comparing 182 paired colorectal cancer and peritumoral tissues, we found that G9a was highly expressed in colorectal cancer (CRC). Overexpression of G9a promoted CRC cells proliferation and colony formation, whereas knockdown of G9a inhibited CRC cells proliferation. Depletion of G9a increased the rate of chromosome aberration, induced DNA double strand breaks and CRC cells senescence. G9a inhibition synergistically increased γH2AX expression induced by topoisomerase I inhibitors and ultimately led to CRC cell death. The findings that down-regulation of G9a triggers DNA damage response and inhibits colorectal cancer cells proliferation may define G9a as potential oncotarget in CRC.

## INTRODUCTION

Colorectal cancer (CRC) is the third most common cancer and the fourth leading cause of cancer deaths worldwide [1, 2]. The World Cancer Research Fund International estimates that nearly 1.4 million new cases of CRC were clinically diagnosed worldwide in 2012, with 102,480 new cases in the United States [3]. Currently, the main treatment for CRC is surgery, radiotherapy, and chemotherapy. However, the 5-year survival rate is less than 40% in less developed countries [4]. Following chemotherapy, more than 50% of patients relapse, and this is associated with metastasis [5]. Advanced stage colorectal tumors are notoriously resistant to chemotherapy and show a very poor 5-year survival [6, 7]. For these reasons, research into the mechanisms behind CRC development is imperative for the identification of novel therapeutic targets.

CRC has been depicted for many years as a prototypic model for the genetic basis of cancer, and is now becoming increasingly cited as an epigenetic model for tumorigenesis. In fact, epigenetic alterations, including DNA methylation and histone modification, are much more prevalent than genetic alterations in the development of CRC [4, 8, 9]. These epigenetic changes that lead to heritable changes in gene expression are now widely used as diagnostic and prognostic molecular markers, as well as novel therapeutic targets.

G9a, which is also known as EHMT2/KMT1C, is a histone methyltransferase (HMT) that contains a Su(var), Enhancer of Zeste, Trithorax (SET) domain, and localizes in euchromatin regions where it catalyzes the mono- and di-methylation of histone H3 Lys9 (H3K9me1/H3K9me2) [10]. In addition to histone modifications, some non-histone G9a substrates such as p53 [11] have been identified. Evidence suggests that G9a is required for the maintenance of the malignant phenotype. Dysregulated expression of G9a was documented in different tumor types, including lung cancer, breast cancer, and hepatocellular carcinoma [12–14]. However, the tumorigenic role of G9a in colon cancer is far from clear. In the present study, we aimed to explore whether G9a plays an important role in the development of CRC.

## RESULTS

### G9a is highly expressed in CRC patient samples

**Figure 1:**
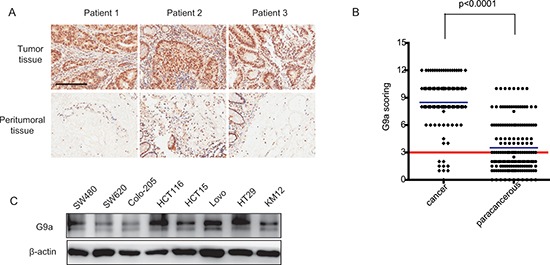
G9a is highly expressed in colorectal cancer **(A)** Representative photomicrographs of G9a immunohistochemical staining from CRC tumors and peritumoral tissues (scale bar 1mm). **(B)** G9a immunohistochemical staining scores for each specimen. Each symbol represents a tumor from an individual patient while the blue lines are group mean scores. The red line is dividing the high and low expression levels of G9a based on the staining scores of immunohistochemical in CRC patients. **(C)** Protein levels of G9a in human CRC cell lines as determined by western blot analyses.

To elucidate a link between G9a expression and CRC, immunohistochemical staining was used to analyze G9a levels in normal and tumor tissues. In all CRC patient tissues, G9a staining was observed mainly in the cell nucleus, with cytoplasmic staining in few specimens. Examination of 182 paired CRC specimens revealed a significantly higher expression of G9a in tumor tissues (*P* < 0.0001; Figure 1A, 1B). We also used a panel of CRC cell lines to profile the expression pattern of G9a. Western blot analysis showed that G9a was expressed in all CRC cell lines tested (Figure 1C). Our data collectively demonstrated that G9a is highly expressed in both clinical samples and CRC cell lines, suggesting a potential role of G9a in maintaining the malignant phenotype of CRC.

### G9a is important for colon cancer cell proliferation *in vitro* and *in vivo*

**Figure 2:**
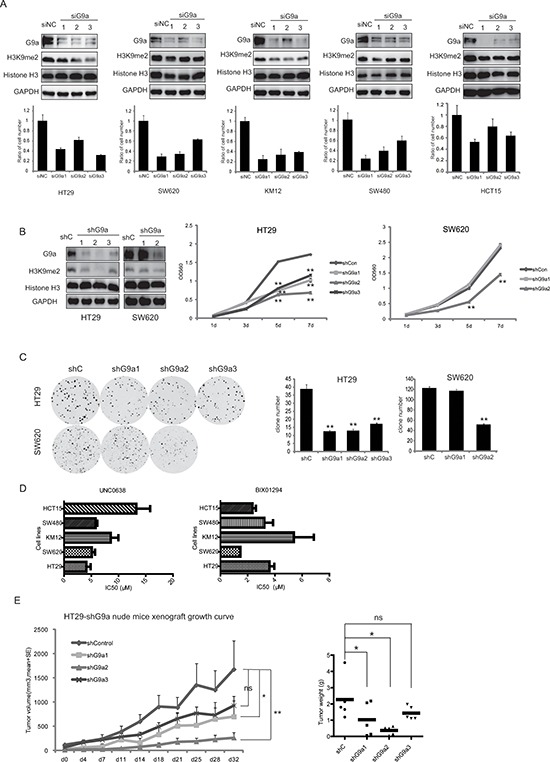
G9a is important to CRC cell proliferation *in vitro* and *in vivo* **(A)** Different siRNAs for G9a reduced G9a expression and delayed CRC cell growth *in vitro*. Histograms show the relative cell proliferation at 120 h after siRNA transfection. Delayed cell growth **(B)** and reduced colony formation **(C)** for HT29 and SW620 cells stably suppressing G9a expression. (**D)** CRC cells treated with different concentrations of the G9a inhibitors UNC0638 and BIX01294. Inhibitory effects of these inhibitors on CRC cell proliferation were measured by the SRB assay (mean IC_50_ values were calculated from at least three independent experiments). **(E)** HT29 cell growth following knockdown of G9a expression and inoculation of cells in nude mice. Data are expressed as the mean ± SE. Tumor weight is also shown. **P < 0.01, *P < 0.05.

**Figure 3:**
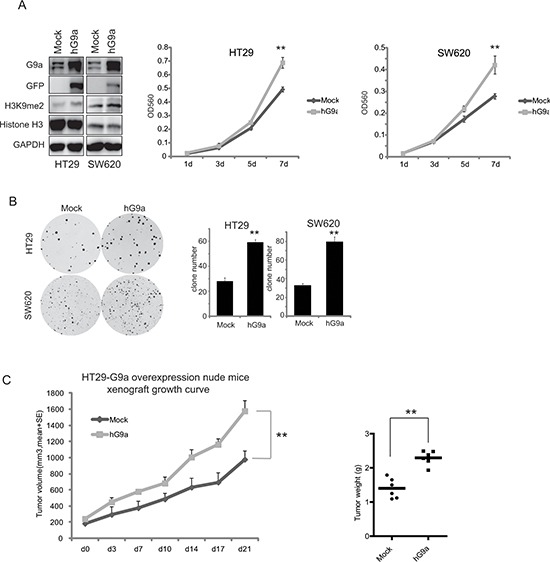
Up-regulation of G9a enhances colon cancer cell proliferation Overexpressed G9a in HT29 and SW620 promotes **(A)** cell growth and **(B)** colony formation *in vitro*. Bars indicate standard deviation of the mean for *n* > 3 determinations. (**C)** G9a overexpression increased the growth of the HT29 cells inoculated in nude mice. Data are expressed as the mean ± SE. Tumor weight is also shown. **P < 0.01.

In order to explore the role of G9a in colorectal cancer development, specific siRNAs were used to knockdown G9a expression in HT29, SW620, KM12, SW480, and HCT15 CRC cell lines. siRNA transfection successfully reduced G9a expression, and consequently suppressed H3K9me2 in all CRC cell lines, whereas no difference in GLP expression was observed. The knockdown of G9a drastically reduced cell growth *in vitro* (Figure 2A). To further assess the effects of G9a expression on cell growth, stable cell lines were generated with limited G9a expression (shG9a1, shG9a2, shG9a3 in HT29, and shG9a1, shG9a2 in SW620) (Figure 2B) and abundant G9a expression (pLEX-hG9a transfected in HT29 and SW620) (Figure 3A). As compared with the parent cells, the cells that stably suppressed G9a expression grew more slowly (Figure 2B), and possessed a reduced capacity for colony formation (Figure 2C). In contrast, overexpression of G9a promoted CRC growth *in vitro* (Figure 3A, 3B). To further substantiate these observations, the G9a specific inhibitors, UNC0638 and BIX01294 were used. These inhibitors significantly reduced CRC cell proliferation, with the IC_50_ values ranging from 1–20 μM (Figure 2D). Our data together suggest that G9a plays a critical role in CRC cell proliferation.

To assess the effect of G9a expression on cell proliferation *in* vivo, CRC cells with different levels of G9a were subcutaneously inoculated in nude mice. All mice developed palpable cancers within 30 days after inoculation, however silencing G9a impaired tumor growth. As shown in Figure 2E, knockdown of G9a expression with shG9a2 most proficiently attenuated HT29 cell growth in nude mice in comparison to the shCon, shG9a1, and shG9a3 groups, with tumor volumes of 266 ± 102 mm^3^, 1678 ± 593 mm^3^, 701 ± 331 mm^3^, and 930 ± 194 mm^3^, respectively on the 32^nd^ day. Alternatively, the tumor volume in the HT29-pLEXhG9a group was statistically larger than that in HT29-pLEXmock, with the tumor volume of the former being 1578 ± 100 mm^3^, while the latter tumor volume was 978 ± 132 mm^3^ on the 21^st^ day (Figure 3C). All these strongly suggest that G9a can regulate the tumor growth of CRC.

### Down-regulation of G9a induces DNA damage response in colon cancer

**Figure 4:**
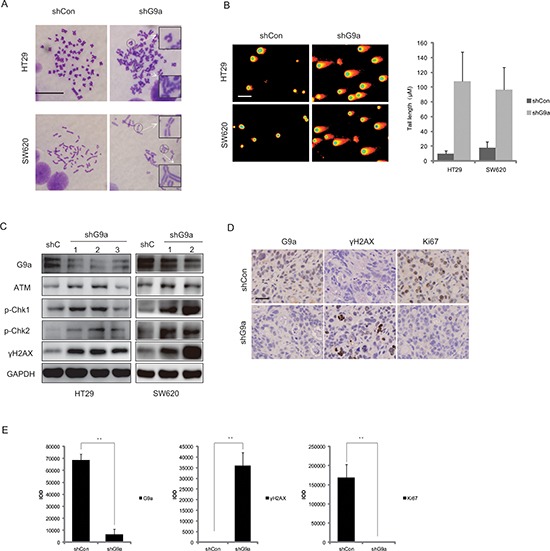
Down-regulation of G9a induces DNA damage in colon cancer **(A)** Karyotype analysis showed that down-regulation of G9a induced chromosome instability (scale bar 10 μM). **(B)** DNA damage as assessed by the comet assay (scale bar 25 μM). **(C)** The protein expression levels for G9a, ATM, p-Chk1 (Ser 317), p-Chk2 (Thr 68), γH2AX, and GAPDH (loading control) following the stable knockdown of G9a in HT29 and SW620 cells. **(D)** Representative immunohistochemical staining images of G9a, Ki67, and γH2AX in xenografts derived from HT29 cells that stably silenced G9a expression (scale bar 25 μM). **(E)** The quantified results by Image Pro Plus based on the immunohistochemical staining in Figure 4D. **P < 0.01.

It has been reported that down-regulation of G9a can induce chromosome instability in cancer cells [15]. By means of karyotype analysis, we found that knockdown of G9a increased the rate of chromosome aberration from 0.55% to 5% in HT29shG9a cells, as compared with cells transfected with shCon (Figure 4A). Given that chromosome instability leads to DNA damage [16], we used a neutral comet assay, a simple, sensitive and rapid method for the detection and quantification of DNA damage [17], to evaluate whether G9a depletion induces DNA double-strand breaks (DSBs). In Figure 4B, the degree of cellular DNA DSBs increased after G9a knockdown in HT29 and SW620 cells, as evidenced by the frequent appearance and expanding volume of comet tails, as well as the shrinkage of comet heads. Furthermore, we found an increased expression of phosphorylated H2AX (γH2AX), which is a well-known marker of DNA DSBs.

Since γH2AX is known to be phosphorylated by members of phosphoinositide 3-kinase related protein kinases (PIKKs) such as ATM (ataxia-telangiectasia, mutated), ATR (ATM and Rad-related kinase), or DNA-dependent protein kinase catalytic subunit (DNA-PKcs) in response to genomic insult [18], we further investigated the potential effect of G9a on these upstream signaling molecules. We found that levels of p-ATM (Ser 1981), p-ATR (Ser 428), ATM, p-Chk1 (Ser 317), and p-Chk2 (Thr 68) increased in G9a-knockdown HT29 and SW620 cell lines as compared to cells transfected with shCon (Figure 4C, Figure S1). Similar results were observed in the *in vivo* studies. We found that Ki67, a hallmark of proliferation, decreased in G9a-knockdown HT29 xenografts, followed by an increased level of γH2AX (Figure 4D, 4E). These studies indicate that suppression of G9a expression triggers DSBs and a robust DNA-damage response in colon cancer.

### Silencing G9a leads to cancer cell senescence

**Figure 5:**
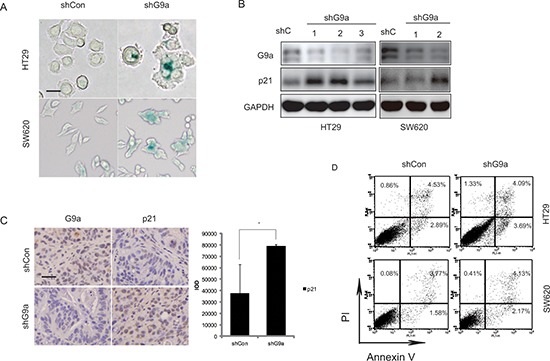
Depletion of G9a results in senescence (**A)** Senescence-associated β-galactosidase expression in HT29 and SW620 cells following stable knockdown of G9a (scale bar 25 μM). (**B)** The p21 protein level detected in HT29 and SW620 cells *in vitro* following the stable suppression of G9a. (**C)** The p21 protein level detected in HT29 shG9a xenografts (scale bar 25 μM). (**D)** Silencing of G9a can not induce CRC cell apoptosis. Percentage of cells in apoptosis was measured by flow cytometry using annexin V and PI staining. The panel in the lower right (annexin V^+^/PI^−^) was considered as cells in early apoptosis. Each condition was studied at *n* ≥ 3. *P < 0.05.

DNA damage often leads to a halt in cell proliferation by triggering apoptosis or senescence, which thereby prevents transmission of harmful mutations onto daughter cells [19, 20]. And γ-H2AX is not only a marker of DNA damage but also a marker of cellular senescence even in the absence of DNA damage [21, 22]. In this regard, we investigated whether G9a knockdown may induce senescence of CRC cells. Since SA-β-gal (senescence-associated β-galactosidase) has been identified as a specific marker for senescent cells [23], HT29 and SW620 cells were then stained with β-galactosidase to determine whether senescence was initiated by G9a depletion-induced DNA damage response (DDR). We found a significant increase of β-galactosidase positive cells when G9a was suppressed in the CRC cell lines (Figure 5A). Moreover, p21, which is considered an inducer of cellular senescence, was up-regulated in these cell lines (Figure 5B). These findings were consistent with those observed in HT29 xenograft tumors that had G9a stably suppressed (Figure 5C). However, knockdown of G9a failed to induce apoptosis, as evidenced by the consistent annexin V+ levels in HT29shG9a cells and SW620shG9a cells (Figure 5D), when compared with shCon cells.

### G9a depletion synergizes with topoisomerase I (TOPO I) inhibitors in CRC cells

**Figure 6:**
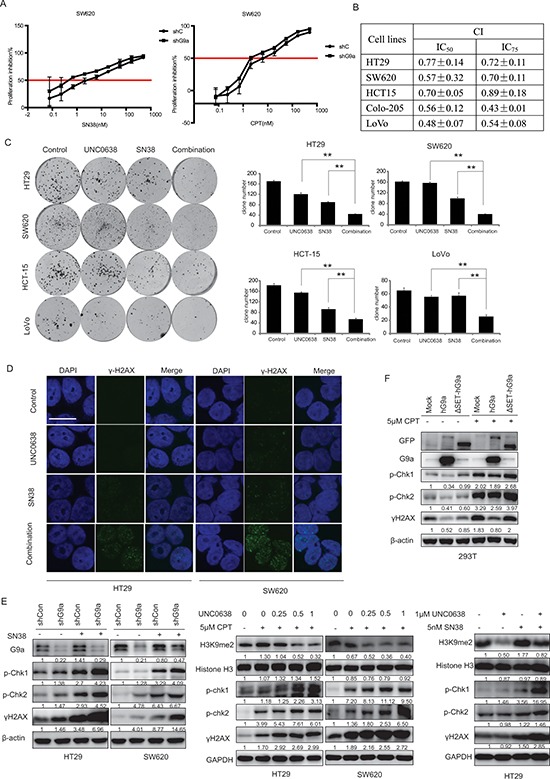
G9a depletion synergizes with TOPO I inhibitors in CRC cells **(A)** SW620 shG9a or shCon cells were treated with different concentrations of SN38 or CPT, followed by subsequent proliferation inhibition assessment using the SRB assay. CRC cells were treated with different concentrations of UNC0638 and/or SN38, and **(B)** cell proliferation was measured with the SRB assay and CIs were calculated, (**C)** colony formation was measured. **(D)** Protein levels of γH2AX were analyzed by microscopy (scale bar 10 μM), and **(E)** protein levels of histone H3, H3K9me2, p-Chk1, p-Chk2, γH2AX and GAPDH were determined by western blot. **(F)** Protein levels of GFP, G9a, p-Chk1(Ser 317), p-Chk2 (Thr 68), γH2AX, and β-actin (loading control) from 293T cells stably transfected with pLEX-mock, pLEX-hG9a, or pLEX-ΔSET-hG9a and treated with the indicated concentrations of CPT. Data are means ± SD, *n* ≥ 3. **P < 0.01.

Combinatorial drug treatment is widely used in cancer therapeutics to combat single-drug resistance, overcome side effects, and increase sensitivity to therapy. Since TOPO I inhibitors are widely used to treat CRC, we investigated whether G9a would potentiate the anticancer activity induced by TOPO I inhibitors. For this, we used two TOPO I inhibitors SN-38 and CPT as combinatorial components. We found that the IC_50_s for SN-38 and CPT were lower in G9a knockdown cells, as compared with parent cells (Figure 6A). We next determined the combination therapy effect by calculating the combination index (CI) using the CalcuSyn program, which is based on the Chou-Talalay method [24]. A CI < 1 indicates a synergistic effect, CI = 1 indicates an additive effect, whereas CI > 1 indicates an antagonistic effect. Our data shows that the TOPO I inhibitors and the G9a inhibitor UNC0638 displayed a synergistic effect in a panel of CRC cell lines, including HT29, SW620, HCT-15, Colo-205, KM12, and LoVo (Figure 6B, 6C). Western blot and immunofluorescence analysis further revealed that either UN0638 or TOPO I inhibitors alone weakly increased the accumulation of γH2AX *in vitro*. However, the combined therapy resulted in a dramatic increase in γH2AX accumulation (Figure 6D, 6E). Consistent with this, increased phosphorylation of p-Chk1 and p-Chk2 was also observed (Figure 6E).

In addition to the above experiments, the 293T cell line was used to generate three stable cell lines: mock (pLEX-Mock), high expression of G9a (pLEX-hG9a), and non-activated mutant-dead G9a (pLEX-ΔSET-hG9a)(Figure S2). We found that p-Chk1 (Ser 317), p-Chk2 (Thr 68), and γ-H2AX levels quickly increased in mock and pLEX-ΔSET-hG9a 293T cells in response to CPT treatment. However, no alteration in p-Chk1 (Ser 317), p-Chk2 (Thr 68), and γ-H2AX were observed with CPT treatment of the pLEX-hG9a cell line (Figure 6F). This suggests that G9a depletion-synergized DNA damage depends on its catalytic activity. Taken together, these results indicate that G9a depletion synergizes with the antitumor activity of topoisomerase inhibitors in colon cancer treatment.

## DISCUSSION

In the present study, we identified for the first time that over-expression of G9a stimulated CRC cell proliferation both *in vitro* and *in vivo*. Moreover, G9a-specific siRNAs, G9a stable knockdown, and G9a specific inhibitors inhibit CRC cell proliferation. Mechanistically, the tumor-promoting effect of G9a appears to bypass its known downstream signaling molecules such as EpCAM [25], PPARγ [26], and Wnt [26, 27] (Figure S3). Instead, G9a depletion increased the rate of chromosome aberration, induced DNA DSBs, and ultimately led to CRC tumor growth arrest. A remarkable increase in γH2AX levels in G9a-knockdown CRC cells was also displayed, followed by the accumulation of p-ATM and p-ATR, which is preferentially activated by DSBs. HMTs' abnormality associated with DDR has been reported these years [28, 29]. Dot1L, which is the only non-SET domain-containing HMT that targets histone H3 lysine 79 (H3K79), can recruit DNA repair component 53BP1 to the sites of damage [30, 31]. EZH2 depletion results in the abrogation of cell cycle G1 and G2/M checkpoints, directing DNA damage response toward predominant apoptosis in both p53-proficient and p53-deficient cancer cells, but not in normal cells [32]. Study of G9a knockout mice showed that G9a was necessary for embryonic development and cell differentiation. It is believed that this embryonic growth defect in G9a-deficient embryonic stem cells may be due to apoptotic cell death but not cell cycle arrest. In that model, chromosomal instability was not observed in the knockout cells [33]. However, it has been reported that an increased number of chromosomes was found in the G9a-knockdown MCF-7 and H1299 cells [13]. The present study provided insight into the possible benefits of down-regulating G9a in colon cancer, with G9a knockdown inducing extensive chromosome instability, which could trigger DNA damage response, cellular senescence, and ultimately tumor growth arrest.

Tumor recurrence following surgery and adjuvant treatment remains a major problem in CRC treatment. Irinotecan (CPT-11) is often used as a first- and second-line chemotherapy for advanced or recurrent CRC. However, only 20–30% of patients show an objective response to CPT-11. There is increasing awareness of an improved outcome in clinical practice when epigenetic inhibitors and cytotoxic agents are combined [34]. Therefore, we also tried to evaluate a combination therapy of G9a inhibitor and TOPO I inhibitors in CRC treatment. As expected, a synergistic anticancer effect of UNC0638 with TOPO I inhibitors were observed in CRC cells. CI values and the significant decline in the colony numbers when the drugs were combined strongly demonstrated that G9a depletion potentiated the SN-38-induced cytotoxicity in CRC cells. Furthermore, the combined therapy synergistically increased γH2AX, caused DNA damage, and led to CRC cell senescence.

In summary, the results obtained in this study indicate that G9a inhibition may be a promising therapeutic target in the treatment of CRC, especially when G9a inhibitors are combined with other cytotoxic agents that have a synergistic effect.

## MATERIALS AND METHODS

### Chemicals and antibodies

UNC0638, CPT were both purchased from Sigma (St. Louis, MO, USA). Senescence β-Galactosidase Staining Kit, RIPA were purchased from Beyotime (Nan-tong, China). The following antibodies were used as primary antibodies: G9a (Cell Signaling, Danvers, MA, USA), ATM (Cell Signaling), p-Chk1 (Ser 317), p-Chk2 (Thr 68), γH2AX (Santa Cruz Biotechnology, Santa Cruz, CA, USA), Histone H3 (Cell Signaling), Histone H3 Dimethyl (K9) antibody (Epitomics, CA, USA), EpCAM (Epitomics), PPARγ (Cell signaling), Wnt6 (Epitomics), Wnt10a (Epitomics), β-actin (Cell Signaling), GAPDH (Cell Signaling), GFP (Santa Cruz Biotechnology), Alexa Fluor 488 Goat Anti-Mouse IgG (H+L) Antibody (Life Technologies, Oregon, USA), Alexa Fluor 594 Goat Anti-Rabbit IgG (H+L) Antibody (Life Technologies).

### Immunohistochemistry

CRC tissue was provided by Shanghai Biochip Company Ltd. The sections consisted of 182 pairs of tumor and matched peritumoral samples. Immunohistochemistry was performed as described previously [35]. Briefly, the EnVision™ Detection Systems Peroxidase/DAB detection kit was used in this experiment. The primary antibody was diluted at 1:1000 and incubated at 4°C overnight. The slides were counterstained with hematoxylin and the tissue image was captured with a digital camera and quantitated by Image Pro Plus.

### Cell culture

The human colon adenocarcinoma HT29, SW620, SW480, HCT15, LoVo, 293T, and 293FT cell lines were purchased from American Type Culture Collection (Manassas, VA, USA). KM12 was from Japan and Colo-205 was from Shanghai Institute of Biochemistry and Cell Biology. HT29 and HCT116 cells were grown in McCoy's 5A medium (Sigma) supplemented with 10% fetal bovine serum (FBS; Gibco, Grand Island, NY). SW620 and SW480 cells were grown in L-15 (Gibco, Grand Island, NY) supplemented with 10% FBS. HCT15 and Colo-205 were grown in RPMI-1640 (Gibco, Grand Island, NY) supplemented with 4.5 g/L glucose, 0.11 g/L sodium pyruvate, and 10% FBS. LoVo was grown in F-12 (Gibco, Grand Island, NY) supplemented with 0.29 g/L glutamine and 10% FBS. KM12 was grown in RPMI-1640 (Gibco, Grand Island, NY) supplemented with 10% FBS. The 293FT Lentiviral Expression System and 293T were cultured in Delbecco's modified Eagle medium (Gibco, Grand Island, NY) supplemented with 10% FBS.

### Plasmids and transfection

pLKO.1-shG9a plasmids were generous gifts from Dr. Jin Jian (University of North Carolina, USA). pEGFP-hG9a, pEGFP-ΔSET-hG9a [36] were obtained from Addgene, and recombined into pLEX to construct pLEX-hG9a, and pLEX-ΔSET-hG9a. These plasmids were transfected into 293FT cells with packaging mix (pCMV-dR8.2 dvpr and pCMV-VSVG) to produce lentiviruses. The stable knockdown and overexpressed G9a cell lines were established as outlined in the Addgene protocols.

### Quantitative real time RT-PCR

Total cellular RNA was isolated with TRIzol (Invitrogen, Carlsbad, CA, USA) and reverse transcribed into cDNA using the PrimeScript™ RT reagent Kit (Takara, Otsu, Shiga, Japan). Real-time RT-PCR was performed on an Applied Biosystems 7500 using SYBR-Green Master mix (Takara) with the following primers: G9a 5′-gccaggccgggaggccctggaa-3′ (sense), 5′-ctccagcctgcagcagcacatg-3′ (antisense); GLP 5′-gccgtggacagcgagccatgcccc-3′ (sense), 5′-ggcaggagccggccatccttgtcgt-3′ (antisense), and GAPDH 5′-gcaaattccatggcaccgtc-3′ (sense), 5′-tcgccccacttgattttg-3′ (antisense). The reactions parameters were: 95°C for 10 min followed by 42 cycles of 95°C for 5 s and 60°C for 34 s. All samples including the template controls were assayed in triplicate. The relative number of target transcripts was normalized to the number of human GAPDH transcripts found in the same sample. The relative quantification of target gene expression was performed with the standard curve or comparative cycle threshold (CT) method.

### Colony formation assay

SW620 and HT29 stable knockdown or overexpressed cell lines were seeded in 6-well plates at the density of 200 cells per well. The cells were cultured for 10–15 days until colonies were visible. Colonies were fixed in 10% formaldehyde and 10% acetic acid at room temperature for 10 min and then stained with 1% crystal violet.

### Sulforhodamine B (SRB) assay

Cells were seeded onto 96-well plates with the same density, and cultured for 1 d, 3 d, 5 d, and 7 d. Cells were then fixed with 10% trichloroacetic acid (TCA), washed with distilled water, and stained with SRB (Sigma, St. Louis, MO, USA) in 1% acetic acid. SRB in the cells was dissolved in 10 mM Tris-HCl and was measured at 560 nm with a spectraMAX190 (Molecular Devices, Sunnyvale, CA).

### Western blot analyses

Cells were collected and lysed in RIPA (Beyotime, Nan-tong, China) supplemented with a protease inhibitor cocktail (Roche, Mannheim, Germany). An equal amount of protein was subjected to SDS-PAGE and transferred to nitrocellulose membranes, and probed with primary antibodies at 4°C overnight. Protein levels were quantified using Image J.

### Comet assay [35]

Slides were pre-coated with 1% normal melting point agarose. About 3 × 10^4^ cells were mixed with 70 μL of 1% low melting point agarose in phosphate-buffered saline (PBS), and rapidly spread onto the pre-coated slides. The slides were immediately placed in cold lysis buffer containing 2.5 M NaCl, 100 mM EDTA, 10 mM Tris (pH 7.5), 1% Triton X-100, 1% INCI, and 10% DMSO at 4°C for 1–3 h. The slides were then placed in the electrophoresis solution for 20 min to facilitate DNA unwinding before electrophoresis was conducted for 20 min at 25 V and 300 mA. After electrophoresis, the slides were washed with PBS and then stained with DAPI. The individual cells were viewed using an Olympus BX51 UV fluorescence microscope(Olympus, Japan).

### Karyotype analysis

Colcemid (final concentration 0.1 μg/mL) was added to culture flasks and incubated at 37°C for 4 h, before cells were trypsinized, collected and centrifuged at 1000 rpm for 6 min. The cell pellet was resuspended gently in hypotonic solution (0.4% KCl:0.4% sodium citrate) to a final volume of 4 mL. The cell suspension was incubated at 37°C for 7 min to swell the cells. This suspension was then centrifuged at 1000 rpm for 6 min. The supernatant was then carefully removed and the remaining cell pellet was fixed in 2 mL of fresh fixative (3:1 v/v methanol and acetic acid) at room temperature for 30 min. Fixed cells were then centrifuged at 1000 rpm for 6 min, and the fixing procedure was repeated three times. Following the last centrifugation, cell pellets were resuspended in 1 mL fixative. A portion of this cell suspension was then placed on a glass slide and stained with Giemsa for ~15 min. More than 100 chromosomes were observed, and the aberrant chromosomes (e.g., visible breaks, dicentric chromosomes, fragmentations, chromosome loss, ring formation, triradial chromosomes, and gaps) were counted. This data was then used to determine the ratio of aberrant chromosomes.

### Immunofluorescence

Cells were grown on chamber slides, fixed with 4% paraformaldehyde, and permeabilized with PBS containing 0.1% Triton X-100. After blocking with 3% bovine serum albumin for 1 h, cells were incubated with primary antibodies overnight. These cells were then washed three times with PBS, and incubated with Alexa Fluor 488 goat anti-mouse IgG (H+L) or Alexa Fluor 594 goat anti-rabbit IgG (H+L) secondary antibodies. Nuclei were visualized with DAPI staining. Fluorescence signals were analyzed using an Olympus Fluorview 1000 confocal microscope.

### Animal studies

Athymic BALB/c 4–6 weeks old nude mice, purchased from Beijing HFK Bioscience Co., Ltd (Beijing, China), were housed in a specific pathogen-free room with a 12 h light/dark cycle at 25 ± 1°C and fed an autoclaved chow diet and water *ad libitum*. All experiments were performed according to the institutional ethical guidelines on animal care and approved by the Institute Animal Care and Use Committee at Shanghai Institute of *Materia Medica*. HT29 and SW620 cells were s.c. injected into the right flank of nude mice at 5 × 10^6^ cells/mouse (six mice per group). Tumor diameters were measured two times per week and tumor volumes (V) calculated using ½ × length × width^2^.

## SUPPLEMENTARY FIGURES


