# Correction: Down-regulation of G9a triggers DNA damage response and inhibits colorectal cancer cells proliferation

**DOI:** 10.18632/oncotarget.27441

**Published:** 2020-01-21

**Authors:** Jie Zhang, Pengxing He, Yong Xi, Meiyu Geng, Yi Chen, Jian Ding

**Affiliations:** ^1^ Division of Anti-Tumor Pharmacology, State Key Laboratory of Drug Research, Shanghai Institute of Materia Medica, Chinese Academy of Sciences, Shanghai 201203, China; ^2^ School of Pharmaceutical Sciences, Zhengzhou University, Zhengzhou 450001, China

**This article has been corrected:** Due to errors in image placement, the representative image of comet assay in group HT29 shG9a for Figure 4B was an accidental duplicate of the picture for SW620 shG9a. The corrected Figure 4 is shown below. The authors declare that these corrections do not change the results or conclusions of this paper.

Original article: Oncotarget. 2015; 6:2917–2927. 2917-2927. https://doi.org/10.18632/oncotarget.2784

**Figure 4:**
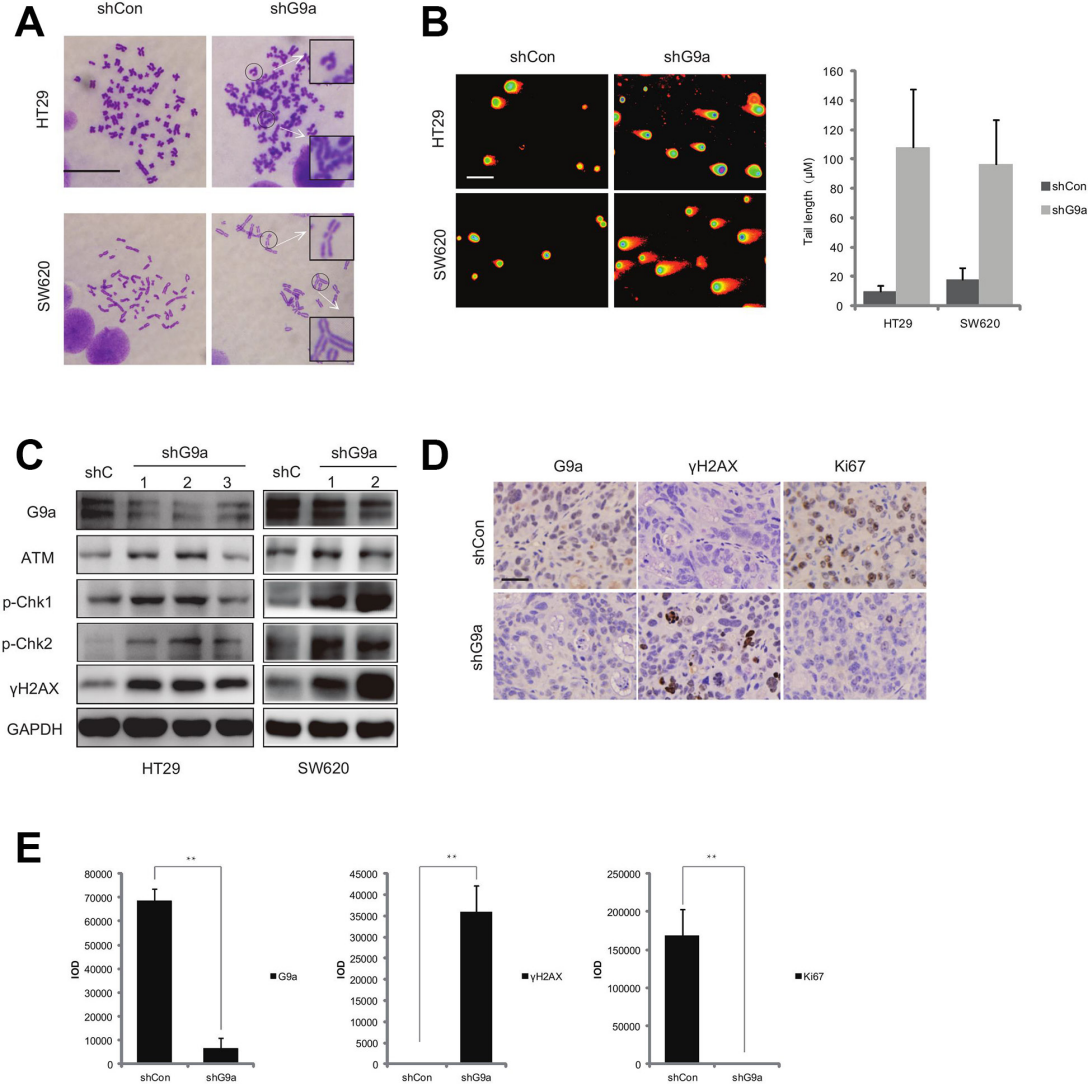
Down-regulation of G9a induces DNA damage in colon cancer.

(**A**) Karyotype analysis showed that down-regulation of G9a induced chromosome instability (scale bar 10 μM). (**B**) DNA damage as assessed by the comet assay (scale bar 25 μM). (**C**) The protein expression levels for G9a, ATM, p-Chk1 (Ser 317), p-Chk2 (Thr 68), γH2AX, and GAPDH (loading control) following the stable knockdown of G9a in HT29 and SW620 cells. (**D**) Representative immunohistochemical staining images of G9a, Ki67, and γH2AX in xenografts derived from HT29 cells that stably silenced G9a expression (scale bar 25 μM). (**E**) The quantified results by Image Pro Plus based on the immunohistochemical staining in Figure 4D. **P < 0.01.

