# Supplementary material for: Down-regulation of G9a triggers DNA damage response and inhibits colorectal cancer cells proliferation
Source: Oncotarget. 2015 Jan 20;6(5):2917–27. doi: 10.18632/oncotarget.2784 (PMC4413627; doi:10.18632/oncotarget.2784)
Supplement: Supplementary file 1 [file oncotarget-06-2917-s001.pdf]

SUPPLEMENTARY FIGURES

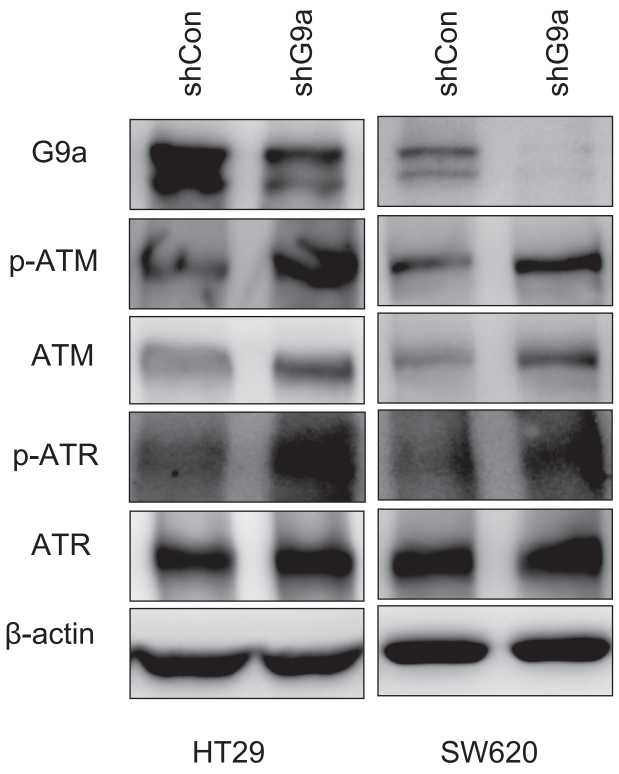

Supplementary Figure S1: Depletion of G9a upregulates the protein level of p-ATM and p-ATR. Protein levels of G9a, p-ATM, ATM, p-ATR, ATR and β-actin (loading control) from HT29 and SW620 cells that stably suppress G9a. Each condition was studied at $n \geq 3$.

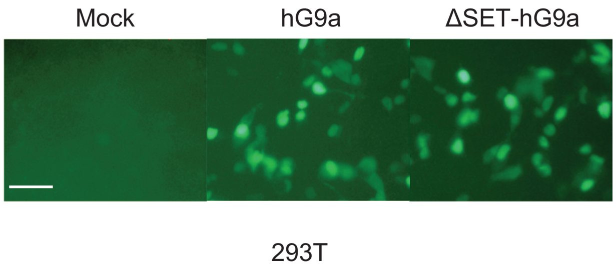

Supplementary Figure S2: The representative pictures of 293T cell after stable transfection with GFP-taged full length or SET domain deleted G9a (scale bar 25 µM).

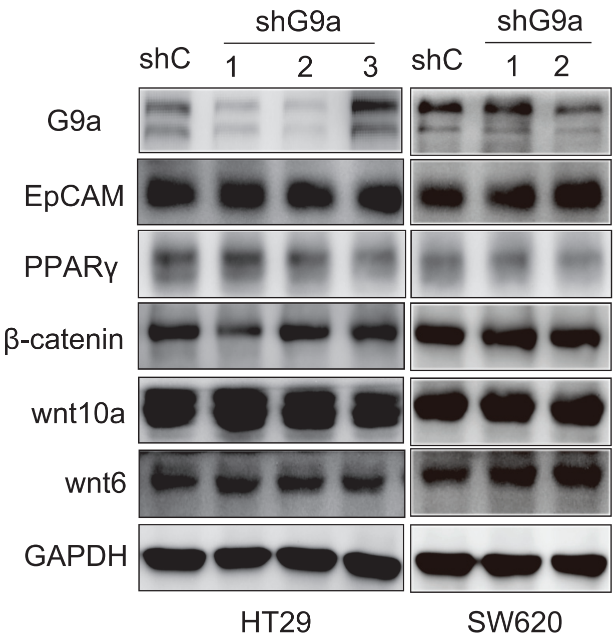

Supplementary Figure S3: Depletion of G9a has no effect on EpCAM, PPARγ, and Wnt expression. Protein levels of G9a, EpCAM, PPARγ, β-catenin, Wnt6, Wnt10a and GAPDH (loading control) from HT29 and SW620 cells that stably suppress G9a. Each condition was studied at $n \geq 3$.
